# The Health and Wellbeing of Indigenous and Tribal Peoples around the Globe: Ensuring and Promoting Best Practice in Research

**DOI:** 10.3390/ijerph19010261

**Published:** 2021-12-27

**Authors:** Kalinda Griffiths, Abbey Diaz, Lisa J. Whop, Joan Cunningham

**Affiliations:** 1 Centre for Big Data Research in Health, University of New South Wales, Sydney 2052, Australia; Kalinda.Griffiths@unsw.edu.au; 2 Menzies School of Health Research, Charles Darwin University, Darwin 0810, Australia; Abbey.Diaz@uq.edu.au (A.D.); Lisa.Whop@anu.edu.au (L.J.W.); 3 Centre for Health Equity, University of Melbourne, Melbourne 3010, Australia; 4 School of Public Health, University of Queensland, Brisbane 4072, Australia; 5 National Centre for Epidemiology and Population Health, Australian National University, Canberra 2601, Australia

## 1. Introduction—Why This Special Issue?

Indigenous and Tribal peoples account for approximately 6.2% of the world’s population, comprising over 476 million people across 90 countries [1]. They have unique cultures, languages, knowledge systems, and traditions, maintain a special relationship with the land, and are guided by their own collectivist worldviews [1]. Indigenous and Tribal peoples across the world continue to be adversely affected by the ongoing impacts of colonization and dispossession, past and present racism and discrimination, socioeconomic disadvantage, and reduced access to services, all of which are manifested in disparities across a range of outcomes [2,3,4]. Research can be a tremendous force for good, provided it reflects the needs and priorities of Indigenous and Tribal peoples, is conducted in ways that empower Indigenous and Tribal people and communities, and privileges Indigenous and Tribal ways of knowing, being, and doing. All too often, this has not been the case [5,6].

In recent years, we have witnessed encouraging developments, such as an increase in research led by Indigenous and Tribal scholars and a gradual shift in how research is conceptualised and undertaken. Our aim for the Special Issue was to showcase best practice in research relating to the health and wellbeing of Indigenous and Tribal peoples, as a way of recognising excellence and encouraging and supporting further advancement. The focus of the Special Issue was on research conducted by, with, and for the benefit of, Indigenous and Tribal peoples. In keeping with our focus on Indigenous and Tribal peoples, our definition of health and wellbeing was a holistic one, incorporating physical, mental, social, emotional, spiritual, and cultural aspects, as well as family and community and connection to land and waters across time. We called for papers that reflected the values of respect, reciprocity, and partnership and that addressed the priorities, needs, and aspirations of Indigenous and Tribal peoples.

The resulting Special Issue includes 31 papers in total, with 21 from Australia [7,8,9,10,11,12,13,14,15,16,17,18,19,20,21,22,23,24,25,26,27], 4 from the United States [28,29,30,31], 3 from Aotearoa/New Zealand [32,33,34], 1 from Canada [35], and 2 from authors in multiple countries [36,37]. This represents a substantial body of research on the health and wellbeing of Indigenous and Tribal peoples, possibly the largest collection ever published.

## 2. Special Requirements for Papers in the Special Issue

In keeping with the ethos of the Special Issue, all submissions were required to address three key points: (a) the nature of the engagement, involvement, and leadership by Indigenous/Tribal people and communities in the project; (b) ethics and governance considerations in relation to Indigenous/Tribal peoples; and (c) whose priorities are reflected in the work. Aside from details about institutional ethics approval, which represents a relatively small component of point (b), this information is not typically reported in academic manuscripts, despite it being central to Indigenous and Tribal peoples’ research paradigms as well as a practical step towards decolonising research. Some authors appeared to struggle with this requirement, perhaps because of a lack of any model to follow. The ways in which the points were addressed varied across the included papers, as described in the following sections.

### 2.1. Engagement, Involvement, and Leadership

The engagement, involvement, and leadership of Indigenous and Tribal people is an essential requirement for ensuring that research is consistent with the rights of Indigenous and Tribal peoples (including the right to self-determination) [38], is culturally safe [39], and reflects Indigenous and Tribal people’s understandings, values, and aspirations and elevates and amplifies their voices [5,39]. This is relevant across the entire research process, from identifying and articulating a research question to designing and conducting the study, making meaning of the results, and communicating and implementing the findings.

Authorship is perhaps the most obvious indicator of engagement, involvement, and leadership. It is a formal and public recognition of an individual’s contribution to the work, although it must be noted that the level of inclusion and influence implied by authorship does not always match the reality. Of the 31 papers in the Special Issue, there were 128 Indigenous/Tribal authors listed, representing 110 individuals after accounting for authors with multiple papers. Indigenous/Tribal authors represented just over half of all authors (51.2%). The number of Indigenous/Tribal authors on an individual paper ranged from 1 to 16 (median = 4), and Indigenous/Tribal authors as a proportion of all authors on a paper ranged from 8% to 100%. Fifteen papers (48.4%) included an Indigenous/Tribal person as first author, and an additional eight had an Indigenous/Tribal person as last author (which is commonly used in health research to indicate senior authorship). At least eight first authors were known to be students, five of whom identified as Indigenous/Tribal. These figures represent substantial involvement, engagement, and leadership by Indigenous/Tribal people across a broad range of career stages.

Engagement, involvement, and leadership beyond authorship are also important. A range of mechanisms across the life of the research project or program, from setting research priorities to ongoing communication to dissemination of results, were described. These included: membership of Indigenous and Tribal people on advisory groups, working groups, steering committees, and governance committees [7,9,10,11,13,14,15,16,23,25,27,30,32,33,34,37]; involvement of community Elders, other community leaders, and Tribal health centres [19,25,28,35]; formal and informal partnerships with Aboriginal Community-Controlled Health Organisations, Tribal health services and other community organisations [8,10,14,19,20,22,25,29,30,31]; hiring and training Indigenous research staff, especially from the relevant community/ies [7,9,10,13,16,18,23,35]; the use of community-based participatory research approaches, co-design, and consumer engagement and involvement [12,19,20,23,25,32,33,35]; and the involvement of existing Indigenous/Tribal expert groups with a remit beyond the project or program, such as an American Indian Data Community of Practice [31] and the International Group for Indigenous Health Measurement [26]. For example, in a project described by Wright and colleagues [19], decolonising research methodologies and co-design were used to develop health service evaluation tools based on First Nations worldviews. This enabled an Aboriginal evaluation framework that was seen as relevant, credible, effective, and meaningful to clients, carers, and mental health services alike. A total of 22 community Elders were involved as co-researchers; 11 of these ‘Aunties’ and ‘Uncles’ (terms of respect used for Aboriginal Elders) were authors on the resulting paper.

Developing relationships of trust between academic researchers and Indigenous and Tribal people, communities, and organisations is critical. Credo and Ingram [30] presented their perspectives on developing successful collaborative research partnerships with Native American communities in Arizona and noted a tension between the time needed to develop and maintain these relationships and the academic pressure on researchers to produce outputs. Overcoming the structural disincentives to invest the time and effort needed to engage meaningfully with Indigenous and Tribal people will require funding agencies and academic institutions to appropriately value this foundational work. The need for flexibility was also highlighted by many authors. For example, Rock and colleagues [29] described their experiences with different approaches to dissemination of research findings to Navajo communities and noted the importance of getting information to people in ways that suit them.

### 2.2. Ethics and Governance

A range of policies, approaches, and processes have been developed to guide the conduct of research on the health and wellbeing of Indigenous and Tribal peoples. For example, the National Health and Medical Research Council (NHMRC), Australia’s primary funder of health and medical research, published updates in 2018 for two complementary guidelines for the ethical conduct of research with Aboriginal and Torres Strait Islander people and communities [40,41]. These documents link into broader national research ethics guidelines and are complemented by the Australian Institute of Aboriginal and Torres Strait Islander Studies’ (AIATSIS) Code of Ethics for Aboriginal and Torres Strait Islander Research 2020 [42]. Funding from the NHMRC is contingent on adherence to these guidelines, which provides a level of structural support for Aboriginal and Torres Strait Islander health research that is conducted ‘in the right spirit, with integrity and with respect for Country and for all living things’ [42] (p. 11). Similar national or regional guidelines exist for research on the health and wellbeing of Indigenous and Tribal peoples in Canada and New Zealand, although the extent to which adherence to these are expected/demanded varies (see, for example, [43,44,45,46]).

In addition to approval by Human Research Ethics Committees and Institutional Review Boards, which are embedded within Western academic institutions, a range of other mechanisms for ensuring appropriate Indigenous and Tribal governance in research were described in the papers included in the Special Issue, such as: approval by a Tribal government, an Indigenous Ethics Committee (e.g., the Aboriginal Health and Medical Research Council’s ethics committee in New South Wales, Australia), or an Aboriginal Community Controlled Health Organisation [7,8,9,10,12,13,14,16,18,19,22,23,25,28,29,31,35]; the use of cultural reference groups and governance committees [11,13,16,19,23,25,27,33,34]; and having formal agreed Terms of Reference and/or Resolutions of Support [10,20,29].

An important element of research governance is ensuring the quality of the research, one aspect of which is using an appropriate research methodology. Several papers noted the use of culturally specific approaches, such as Kaupapa Māori research [32,34] and Indigenist research methods [8,10,13,16]. For example, Adcock and colleagues [34] used Kaupapa Māori research principles [5,47] to examine the experiences of preterm birth and neonatal intensive care for families of Māori infants. Importantly, the research team sought information about both experiences and the cultural meanings ascribed to those experiences, which together can inform appropriate service transformation. Garvey, Anderson, and colleagues [13,16] conducted a large multi-phase study to identify and understand the foundations of wellbeing for Aboriginal and Torres Strait Islander people, from their own perspectives and in their own words. Using an approach based on the core principles of Rigney’s Indigenist research methodology [48], the study team conducted Yarning Circles with hundreds of Aboriginal and Torres Strait Islander people from across Australia. The data were analysed by an Indigenous researcher group and an Indigenous Project Advisory Group using a collaborative yarning process to ensure the cultural coherence of the resulting conceptual model.

Some authors specifically mentioned aspects of data governance and data sovereignty, such as Tribal/community ownership of data, approval of manuscripts for publication, establishment of a data governance committee, and reference to specific data sovereignty principles [10,11,20,26,27,28,30,35]. For example, Ward and colleagues [35] sought to understand the role of land in the wellbeing of Labrador Innu people. Their work used Innu knowledge and ways of knowing through community-based participatory research and was guided by OCAP^®^ principles [44] relating to the ownership, control, access, and possession of First Nations data. Griffiths and colleagues [26] conducted a systematic review of Indigenous data governance in health research internationally. Key aspects identified in the review were Indigenous governance, institutional ethics, socio-political dynamics, data management and stewardship, and overarching influences including human rights, capacity, and funding.

### 2.3. Whose Priorities

Research is inherently political; what gets researched and how and by whom research is conducted are influenced by a range of factors such as power and control as well as social values, norms, and beliefs [49]. This has meant that the priorities of Indigenous and Tribal peoples have not always been the impetus for research about their health and wellbeing [50]. This has important implications for the usefulness of research for improving policy and practice and, ultimately, outcomes.

Studies in the Special Issue arose in a variety of ways. Some were investigator-driven, based on the results of previous research and/or discussions with various stakeholders over many years [7,9,11,13,15,16,17,18,21,25,26,29,32,36,37]. Other studies reflected the priorities of government agencies, either through alignment with articulated strategies or through commissioned work [24,25,33]. Many studies reflected the expressed needs and priorities of Indigenous and Tribal communities, either directly or through community organisations, Tribal governments, and/or Indigenous/Tribal advisory committee members [7,8,10,12,14,19,20,22,23,28,30,31,33,34,35].

The question of whose priorities were being addressed was central to some of the research reported in the Special Issue. For example, Bennett-Levy and colleagues [25] presented a case study in which a top-down, government-initiated digital mental health program was shifted through community-based participatory research to a ground-up, community-guided process that better met community needs by focusing on social and emotional wellbeing more broadly. Cullen and colleagues [8] described the implementation of a model aimed at enabling trauma- and violence-informed care through decolonising interagency partnerships. This work was led by Aboriginal Community-Controlled Health Organisations who were trying to work with government agencies who failed to understand the profound impact of trauma for Indigenous clients.

One especially important signal of priority is funding. Doing research requires resources, both human and otherwise, and lack of resources can be an important impediment to conducting the right research by the right people in the right way. Although we did not specifically request information about funding, this was routinely provided by authors in the Funding section of the manuscript template. For the papers included in the Special Issue, funding sources varied widely. Some papers reported no external funding for the project, while others reported project funding from highly competitive grant bodies and/or from universities, local health districts/boards, state and national government departments, charitable trusts, and non-government organisations. In addition, there was substantial ‘people support’ for many authors, including fellowships and scholarships from national research funding bodies, universities, and other organisations. Some funding bodies have explicitly made research on the health and wellbeing of Indigenous and Tribal peoples a priority in recent years. For example, in Australia, the NHMRC has earmarked at least 5% of its research budget for Aboriginal and Torres Strait Islander health research [51]. This target was reportedly met in 2008 and subsequently exceeded [52], although it must be noted that not all of the funds have been awarded to projects led by Aboriginal and Torres Strait Islander researchers. In New Zealand, the Health Research Council (HRC) has a range of mechanisms to advance Māori knowledge, resources, and people and to support Māori sovereignty in research. For example, since 2019 all research proposals submitted to the HRC are scored on their potential to contribute to Māori health advancement, and grants are available to support communities to undertake research that meets their specific needs [53]. Initiatives such as these send a clear signal to the research community about the importance of work in this area.

## 3. Improving Research Practice

Achieving the vision of having ‘the right people doing the right work in the right way’ requires both incremental and transformative change.

While incremental change represents progress, it is often uneven, and the pace can be frustratingly slow. Two papers in the Special Issue argued for a more transformative approach to change in health and wellbeing research. Duke et al. [17] proposed a new Culturally Adaptive Governance framework designed to address power imbalances and improve the equity of outcomes in Indigenous health research. The framework focuses on what the authors describe as real-world ethics, adaptive governance, and critical allyship. The importance of Indigenous governance, including consideration of place, people, relationships, and process, was identified as a prerequisite for ethical conduct and practice.

Watego and colleagues [21] described their recently funded program of work to develop Indigenist Health Humanities as a new field of enquiry and research, one committed to Indigenous advancement with Indigenous intellectual sovereignty at its core. They argue that poor health must be seen as a function of ongoing colonisation rather than as a product of Indigenous deficit, and they highlight the critical importance of understanding how race operates in society and how the impacts of racism are embodied. They stress the need for researchers to ‘shift the gaze away from Indigenous incapability to consider how institutions, structures, systems, and processes operate to undermine Indigenous health and wellbeing’ [21] (p. 4).

While we believe that transformative change is ultimately required to eradicate the inequities in health and wellbeing experienced by Indigenous and Tribal peoples around the globe, we must not simply go about ‘business as usual’ while we wait for radical overhaul to occur. Instead, we must continually improve our research practices within existing systems and hold researchers and institutions to an ever-higher standard of practice while also working to achieve transformative change. In addition to a growing number of guides on ethics and governance such as those mentioned above, recent attention has been given to how best to report [54] and evaluate [55] research with a focus on Indigenous and Tribal peoples. For example, Gopalani and colleagues [31] described the development and implementation of an HPV vaccination survey in the Cherokee Nation using the Consolidated Criteria for Strengthening the Reporting of Health Research involving Indigenous Peoples (CONSIDER) statement, which covers eight domains: governance; relationships; prioritisation; methodologies; participation; capacity; analysis and findings; and dissemination [54]. In Australia, an Aboriginal and Torres Strait Islander Quality Appraisal Tool has been developed to assess the quality of research from an Indigenous perspective. The 14-point check list covers aspects such as Indigenous research leadership and governance, community engagement, whose priorities are reflected, and whether the approach is strengths-based [55]. The ideas behind these guides are not new. Two decades ago, for example, researchers in Aotearoa/New Zealand were urged to engage in critical self-reflection of how their work addressed key considerations, including initiation, benefits, representation, legitimation, and accountability [56]. In order to achieve positive incremental change, researchers, academic institutions, and research funders alike must take all necessary steps to ensure that research relating to the health and wellbeing of Indigenous and Tribal peoples adheres to the principals, processes, and practices articulated in documents such as these.

## 4. Next Steps

Moving away from colonial practices of research to those embodying Indigenous ways of knowing, doing, and being is urgently needed to maximise the benefits of health and wellbeing research for Indigenous and Tribal peoples around the globe. This Special Issue has highlighted examples of how this has been and can be achieved. However, the structural barriers to doing so and the potential for high personal and academic costs must be acknowledged. There is a critical role for funders, institutions, and research teams in ensuring ‘the right people are doing the right work in the right way’ by recognising, valuing, and supporting the principles, processes, and practices that underpin high quality, culturally safe, and priority-driven research over the metrics that typically define success and impact from a colonial perspective. This is critical to advancing the self determination of Indigenous and Tribal peoples within and beyond research and to supporting the pursuit of transformative change.

In the meantime, as part of shifting the culture and changing expectations about research on the health and wellbeing of Indigenous and Tribal peoples around the globe, we urge journals to require authors to explicitly address: (1) the nature of the engagement, involvement, and leadership by Indigenous/Tribal people and communities in the project; (2) ethics and governance considerations in relation to Indigenous/Tribal peoples; and (3) whose priorities are reflected in the work. We also strongly encourage journals to include among their recommended research and reporting guidelines those that have been designed specifically for Indigenous and Tribal peoples’ health research (such as the CONSIDER framework [54]), to ensure the appropriate conduct and reporting of research in this area. These simple steps could help to promote incremental improvement in research practice and enhance the value of the research while we work towards transformative change.
